# Analytical and Numerical Models of Thermoplastics: A Review Aimed to Pellet Extrusion-Based Additive Manufacturing

**DOI:** 10.3390/polym13183160

**Published:** 2021-09-18

**Authors:** Alessio Pricci, Marco D. de Tullio, Gianluca Percoco

**Affiliations:** Department of Mechanics, Mathematics and Management (DMMM), Polytechnic University of Bari, Via E. Orabona 4, 70125 Bari, Italy; marcodonato.detullio@poliba.it (M.D.d.T.); gianluca.percoco@poliba.it (G.P.)

**Keywords:** pellet extrusion, industry 4.0, polymer melting modeling, single-screw extrusion, additive manufacturing

## Abstract

Recent developments in additive manufacturing have moved towards a new trend in material extrusion processes (ISO/ASTM 52910:2018), dealing with the direct extrusion of thermoplastic and composite material from pellets. This growing interest is driven by the reduction of costs, environmental impact, energy consumption, and the possibility to increase the range of printable materials. Pellet additive manufacturing (PAM) can cover the same applications as fused filament fabrication (FFF), and in addition, can lead to scale towards larger workspaces that cannot be covered by FFF, due to the limited diameters of standard filaments. In the first case, the process is known as micro- or mini-extrusion (MiE) in the literature, in the second case the expression big area additive manufacturing (BAAM) is very common. Several models are available in literature regarding filament extrusion, while there is a lack of modeling of the extrusion dynamics in PAM. Physical and chemical phenomena involved in PAM have high overlap with those characterizing injection molding (IM). Therefore, a systematic study of IM literature can lead to a selection of the most promising models for PAM, both for lower (MiE) and larger (BAAM) extruder dimensions. The models concerning the IM process have been reviewed with this aim: the extraction of information useful for the development of codes able to predict thermo-fluid dynamics performances of PAM extruders.

## 1. Introduction

Industry 4.0 has introduced new business models focused on consumers and product customization. As a natural consequence, both the quantity of the service provided and the added value have increased.

The main points of Industry 4.0 revolution are automation, interoperability, and transparency of information [1], combined with an underlying ethics conscious of the need for processes with low environmental impact.

Additive manufacturing (AM) plays a key role in the Industry 4.0 scenario, leading to the manufacturing of a physical three-dimensional object starting from a computer-aided design (CAD) model.

Moreover, AM allows substantial savings in terms of logistic costs, giving the opportunity to perform 3D printing once the printing file has been acquired, wherever the 3D printer is located.

AM satisfies the growing need for product customization, leading to the development of functional, flexible, and efficient parts and assemblies. Nevertheless, the most common additive manufacturing process, namely the fused filament fabrication (FFF), is characterized by a multitude of limitations.

FFF is an AM process which consists in manufacturing products starting by extruding a polymeric material given in the form of a filament, which is then gradually molten through an extrusion system. Initially, raw filament is pushed down by a pair of wheels. Then, the filament reaches the heated part of the extrusion system. A gradual melting process begins because of the heat generated by a series of resistors. Finally, the fused filament is extruded through a nozzle and it is deposited into a temperature-controlled environment, usually an oven.

The process continues through a layer-by-layer deposition until the final object has been built.

In this context, pellet additive manufacturing (PAM) has gained the interest of the research and industrial community in the last years.

In reference to PAM, a distinction must be made with respect to the printable volumes. For low printable volumes, the expression MiE is used, while for larger ones the process is called BAAM.

FFF provides an effective benchmark for MiE, because of the similarity in terms of printable volumes. Nevertheless, the deposition process is different.

**Figure 1:**
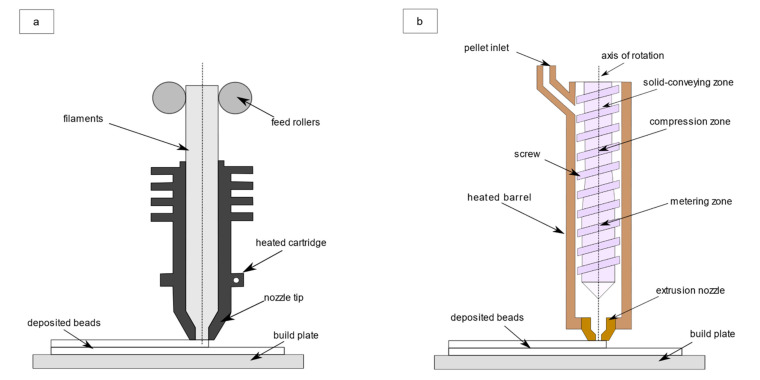
(**a**) Perspective view of FFF process; (**b**) front view of the complete extrusion system in PAM.

In PAM, the material is initially given in the form of pellet and it is conveyed through a hopper in a single-screw extrusion system. In Figure 1a, the FFF process is represented, while in Figure 1b an insight into a PAM extruder is given.

Because of the load exerted both by the rotating screw and gravity, the pellet drops downstream in PAM extruders screw vanes, following a helical path. The pellet is gradually heated until it reaches the melting point (although there is a softening zone instead of a sharp melting point for amorphous polymers).

The low environmental impact, lower costs, and the possibility to combine pellets of different material in MiE are the main advantages of the well-established FFF process.

On the other hand, the rate of deposition in BAAM is much higher than in FFF and MiE; in fact, in FFF, there is a limitation in diameter for the filaments that can be printed. In addition, the extrusion nozzle dimensions for both FFF and MiE are much smaller than that in BAAM.

External heat is provided by several heated sections, namely the barrel. The barrel is the external envelope which contains the extrusion screw. Finally, the molten polymer reaches the extrusion nozzle and is deposited as a filament whose continuity is primarily related to the pellets’ dimensions and hygroscopic content [2]. The first can lead to clogging and stall of the screw or to a non-uniform deposition [3]. The latter can be minimized by subjecting the pellet to controlled thermal cycles.

The FFF process suffers from several limitations, such as:

- filament is expensive, if compared to the same material given in the form of pellet,
- limited range of materials for filament deposition,
- buckling of the filament if its feeding speed is too high,
- high energy consumption of conventional filament extrusion systems,
- high environmental impact of the filament,
- low printing volumes, and
- low productivity.

The weaknesses of the FFF process become strengths for pellet extrusion-based systems. Indeed, cost related to the use of pellet as raw material can be reduced by a factor 20× if compared to the same material given in the form of filament [4].

The range of printable materials is wider by far, because not every polymeric material can be supplied in the form of filament, while it can be purchased in granular or pellet form.

By using pellet, there is no buckling, which is a problem strictly related to the push of the solid filament inside FFF heating chamber.

**Figure 2:**
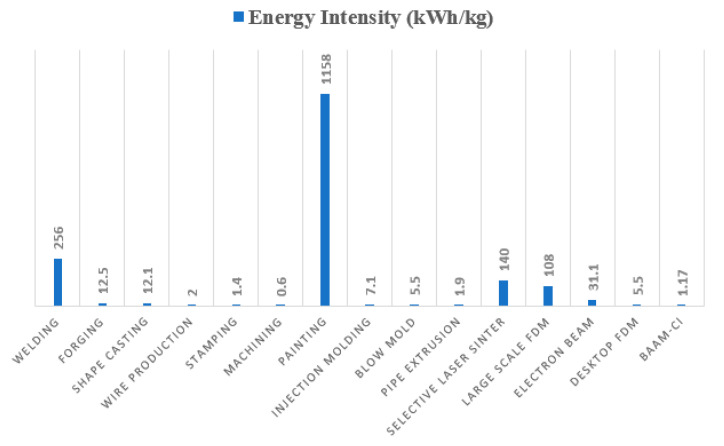
Energy requested for different manufacturing processes—reprinted from [5].

It has been demonstrated (Figure 2) that about 95% of the energy consumption of a conventional manufacturing 3D printing process is related to the heating of the temperature-controlled environment where the deposition process takes place [5]. In the case of both advanced FFF and PAM processes, it is possible to overcome this problem by adding carbon fibers as reinforce material. This solution increases thermal conductivity and reduces thermal expansion coefficient, allowing for a very good bond of the filaments deposited without the need for an oven [5].

It has been stated that Industry 4.0 principles look for low environmental impact manufacturing processes. The impact related to pellet disposal is lower than for filament. In addition, while in FFF there are two melting steps, namely melting of pellet material to create filament and then the melting process of that filament in the FFF machine, in PAM there is only the pellet melting process, without the need to produce filaments as intermediate products.

These advantages are shared by BAAM and MiE because they are not related to extruder dimensions.

In fact, the main difference between BAAM and MiE lies in the larger size of the former.

Because of the higher extruder diameter, the printing volume in BAAM grows significantly; in general, the printing volume in FFF and MiE lies in the range 0.03–0.3 m^3^ [4]. The printing dimensions stated by the same authors for a BAAM apparatus enables to manufacture products up to 6 m long, 2.4 m deep, and 1.8 m high, namely about 10 times more than in FFF and MiE.

Productivity increases, turning to BAAM, because of a growth up to 200× of the mass flow rate [4]. This large increase in mass flow rate is possible also because of nozzle dimension; while in FFF and MiE, nozzle diameter is typically of 0.03 cm [3], in BAAM it is 0.8 cm, which is around 30 times larger [6].

All these advantages call for models, analytical and/or numerical, that can be used to perform accurate analysis of pellet extrusion-based processes and that can account for different dimensions involved in BAAM and MiE.

Clearly, PAM processes are not free of disadvantages, especially when it comes to BAAM, because of the bigger dimensions of the final products and the higher deposition rate. In fact, it can be stated that:

- BAAM is a single material process and it could be difficult to remove support structures without proper post-processing; part orientation becomes more critical than in the case of FFF.
- The larger bead size reduces the printable resolution of the features that can be made by BAAM; the nozzle dimension which gives the best compromise between resolution and printing speed must be chosen.
- BAAM exhibits poor surface finish caused by the larger bead dimensions, if compared to FFF.
- The higher temperatures, larger size, and both bead width and height develop higher internal stress and warping.
- It is more difficult to cool down the layer deposited by BAAM because the deposition rate is higher than in FFF.

To the best of authors’ knowledge, no specific model describing the complete extrusion dynamics inside pellet-based AM processes, such as BAAM and MiE, has been published.

However, it must be recognized that a well-known manufacturing process, namely injection molding (IM), shares several aspects with PAM. It is worth noting, however, that some of the main assumptions applied in modeling the IM process cannot be used in the transition to a mathematical theory for MiE, mainly because of the smaller size of the extrusion system used (i.e., the 28 mm initial screw diameter in [7], in contrast with the 63.5 mm of IM machine studied in [8,9,10]).

It is also expected that smaller dimensions have an influence on melting performances, an aspect that will be addressed in Section 2.2. Furthermore, while in IM there is the injection phase of molten polymer in the die through an axial movement given to the screw, this dynamic is completely absent in BAAM and MiE, where there is no longer a die but a nozzle, similarly to FFF (Figure 1). 

The structure of this review paper can be summarized as follows: in Section 2, we give a general introduction to the internal structure and polymer melting dynamics for an IM machine. Then, in Section 2.1, solid-conveying zone is discussed both analytically (Section 2.1.1) and numerically (Section 2.1.2). The distinction is justified by the purpose to give a better understanding in the framework of the different zones. The same distinction is made for compression and metering zones, respectively in Section 2.2 and Section 2.3.

## 2. Models for the Material Extrusion

A single-screw extruder includes three important areas for material extrusion; these are the solid-conveying zone, compression zone, and metering zone. This architecture is common to the IM, BAAM, and MiE processes.

**Figure 3:**
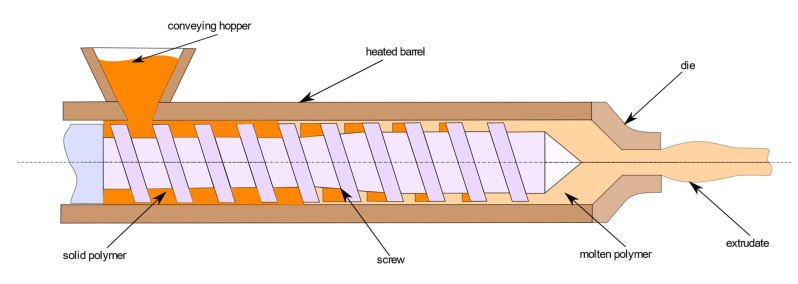
Scheme of the extrusion process in IM.

Before moving to outline the analytical and numerical models which have been proposed in literature for IM process modeling, it is important to pause on the internal structure of a single-screw extruder, which is represented in Figure 3.

In general, the extrusion system consists of one or more screws housed in a barrel; the assembly made of both screws and barrel is joined on the one hand to the hopper for the supply of raw material and on the other hand to the die (IM process) or to the extruding nozzle (PAM processes).

In the following, single-screw extruders will be considered, where the material moves downstream in the channel between barrel and screw. These two elements are separated by a radial clearance where both leakage flow and high viscous thermal dissipation occur.

Material is initially conveyed in the solid-conveying zone in the form of pellet or granules through a hopper. Moving downstream from the hopper there is the screw, enclosed by the barrel.

Then, mass flow rate passes through geometrical vanes between screw and barrel, usually characterized by a constant cross-section in the solid-conveying zone.

From an experimental, analytical, and numerical point of view, pressure increases as the flow rate moves downstream. Because of this pressure, there is the compaction of polymeric material, which reduces the air gaps between pellets.

The surrounding barrel is usually heated in different sections at different temperatures. Through thermal conduction, heat reaches the polymeric pellet material and, when solid temperature exceeds melting point, a gradual melting process begins. Melting is due to both conduction and heat generated by viscous dissipation; this process takes place in the compression zone. This is characterized by a screw channel cross-section usually tapered; the initial to final channel depth ratio is referred to as compression ratio.

Polymer melts according to a certain melting mechanism (Maddock, Lindt, or Klenk), which can change under different operating conditions. Further details on these mechanisms will be given in Section 2.2.

Finally, the material, which now is only at molten state, is transferred towards the die, in the case of an IM machine, in the so-called metering zone. Generally, this zone is characterized by a constant cross-section, as in the case of the solid-conveying zone. The operative point of an IM extrusion process is identified by the intersection of the operating characteristics of both die and screw, which are the pressure-flow rate relationships for the two elements.

In the following, the main works presented in the literature for IM process modeling are reviewed, underlining what assumptions cannot be applied in the extension of those models to BAAM and MiE.

### 2.1. Solid-Conveying Zone

#### 2.1.1. Analytical Modeling

**Figure 4:**
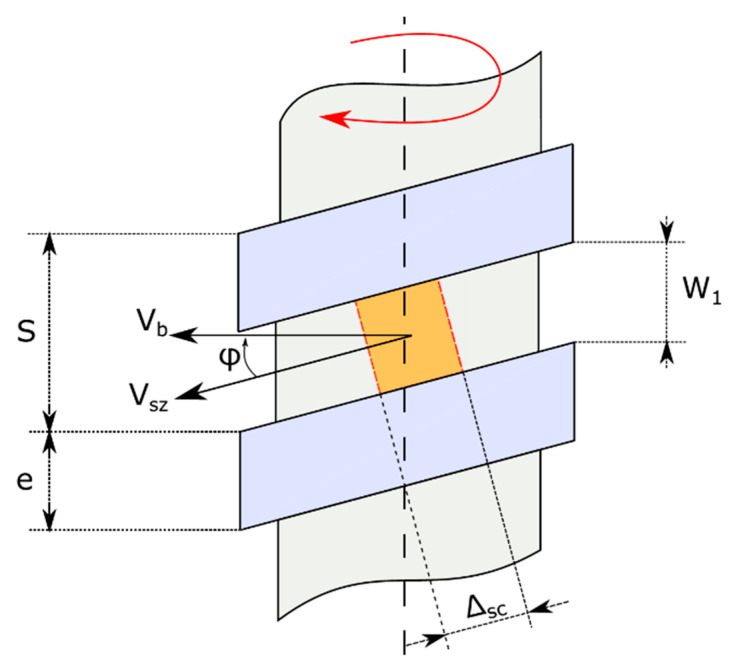
Solid conveying kinematics and geometry; W_1_—channel width at mean diameter, S—screw lead, e—flight width, φ—helix angle, Δ_sc_—computational step in downstream direction, V_b_—barrel velocity, V_sz_—solid body velocity.

The first pioneering work aiming at analytically describing the solid-conveying zone is due to Darnell and Mol [11], who described the polymeric material as a whole and rigid body during the entire conveying process (Figure 4). In their model, as in many works derived from it, it is assumed that there are no air gaps and the mutual sliding of pellet particles is also nullified because of the former assumptions.

In the model, the rigid motion of the whole body takes place under the influence of the friction forces which are generated at the contact interface between the polymer and metal surfaces of screw and barrel. This is also known as the solid friction model.

Another study [12] states that polymer melts rapidly and adheres to walls when the temperature exceeds melting point. This theory is based on experimental observation. The same author in [13] suggests a model where the motion of the solid material happens because of thin molten layers located on both barrel and screw surfaces. This model is known as the viscous drag model.

Early models, e.g., that proposed in [11], include the assumption of an isotropic pressure, which is not the case even in the solid-conveying zone.

A way to consider the anisotropic pressure distribution, which is expected to characterize the solid body during its motion, is to replace the usual isotropic pressure with a compressive stress system with screw root, flights, and barrel surface pressures which are proportional to that along downstream direction [14].

Moreover, the assumption of isothermal conditions is frequently used, which is in contrast with the experimental evidence; indeed, on the contact surface between polymer and metal, there is a large heat exchange, which results in anisotropic distribution of temperature in the solid polymer.

However the assumption of isothermal process is locally justifiable in the models where the calculation of solid properties in solid-conveying zone is performed by dividing the channel in the downstream direction in a series of little increments; for each step, it is possible to evaluate a mean temperature and the friction coefficients at screw and barrel surfaces at that given temperature, obtaining more accurate results if compared to the use of mean values for the whole conveying process. A similar study has been proposed in [15]; by performing a force balance for each segment of solid material, the pressure along the screw axis as a function of geometric screw parameters can be obtained. Pressure in this model is anisotropic and the channel cross-section can be also variable.

In [16], there is a similar computation of the temperature distribution in the solid body, which allows for an accurate calculation on the one hand of the friction coefficient and on the other hand of the axial position where the melting process begins.

In [17], it is assumed that the solid body is pushed upwards, orthogonally to the screw, by the flights. This vertical thrust is assumed to be proportional to the frictional force exerted by the pellets as they move in relative motion with respect to the barrel.

Analytical models described so far do not consider either gravity or centrifugal forces, which can also play a significant role in solid-conveying zone of IM, BAAM, and MiE screw-extruders.

In particular, Darnell and Mol find an exponential relationship between the locally isotropic pressure and axial position along the machine, finding a pressure profile which is independent of the real arrangement of the extrusion system. Lovegrove in [18] modified Darnell and Mol’s theory by adding a pressure term related to the two volume forces mentioned earlier, material being conveyed and the real system arrangement, namely horizontal or vertical. Another advantage of Lovegrove’s theory is that it considers local pressure as anisotropic through the same approach suggested by Schneider in [14]. Considering local pressure as anisotropic and accounting for the effect of a vertical arrangement are key points for an analytical modeling of solid-conveying zone, both in the case of BAAM and MiE.

Nevertheless, all these studies start from the assumption of considering the material as a whole body in rigid motion, which is not the case in real extrusion systems. The only way to consider the complex dynamics of pellets, determined by their interaction, is to turn to numerical models.

#### 2.1.2. Computational Modeling

There are different ways to deal with the motion of solids in screw vanes of the feeding zone. A first approximation is generally given by considering the solid body as a fluid with a very high viscosity and treating it through computational fluid dynamics (CFD) techniques.

In [19], the problem of the motion of the solid polymer was handled by assuming an incompressible fluid of high viscosity, performing a finite difference study in non-isothermal conditions and with a wall-sliding motion proportional to the shear stress.

The assumption of dealing with the solid as a fluid with very high viscosity was also applied for modeling the other zones of extrusion process, namely the compression and metering zones, in CFD. For the last turns of solid-conveying zone, this approach was followed in [8,9,10,20].

A limit which is shared by the described analytical models and this first class of computational ones is that they do not consider the internal friction which arises because of the mutual sliding of the involved pellets. Internal friction is disregarded because of the assumption of a whole, compact body in the solid-conveying zone, subject to a rigid motion in the downstream direction.

To overcome this limit, the discrete element method (DEM) is one of the best choices, but the drawback is that its accuracy is strictly related to an accurate experimental characterization of the material being extruded. Fundamental properties which must be set up, regardless of the DEM software used, are the coefficient of restitution, both internal and external friction coefficients, and the most appropriate contact model [21].

**Figure 5:**
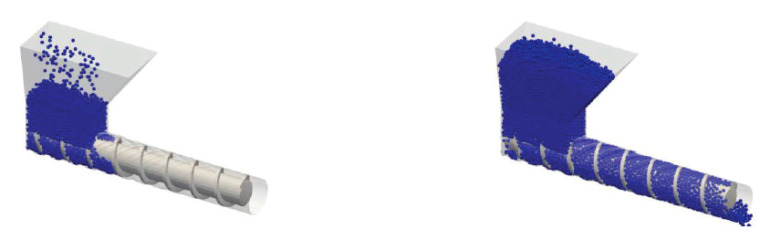
Overview of a DEM simulation of spherical particles—reprinted with permission from [22], with the permission of AIP Publishing.

In all the DEM models, the interaction between single particles is considered (Figure 5) by determining for each of them both normal and tangential actions exchanged with the neighbor particles, as well as the gravity effect. Then, dynamics equations are solved, and acceleration is determined for each particle. Finally, velocity and position of all pellets are determined by integration and this information is used for the next time step.

In contrast to the finite element method (FEM), finite volume method (FVM), and finite difference method (FDM) usually implemented for the numerical solution of governing equations in fluid dynamics problems, in DEM, the particles are no longer linked by a rigid mesh [22].

Another drawback of DEM lies in the high time required to perform computations, mainly because of the low time step needed for obtaining an accurate simulation of momentum exchanged between pellets.

Various DEM three-dimensional models have been developed in order to simulate both the hopper and conveying zones of a single-screw extruder used for IM [22,23,24,25,26].

The experimental investigations lead by Celik in [22] show how flow rate and pressure field depend in a great extent on the shape of particles used in DEM simulations.

Moreover, material and pellet shape also affect temperature field and the force exchanged by the particles [21].

In [27], DEM is applied to predict velocity profile in both downstream and cross direction, as well as coordination number, finding numerical results which show a good agreement with the experimental ones.

**Figure 6:**
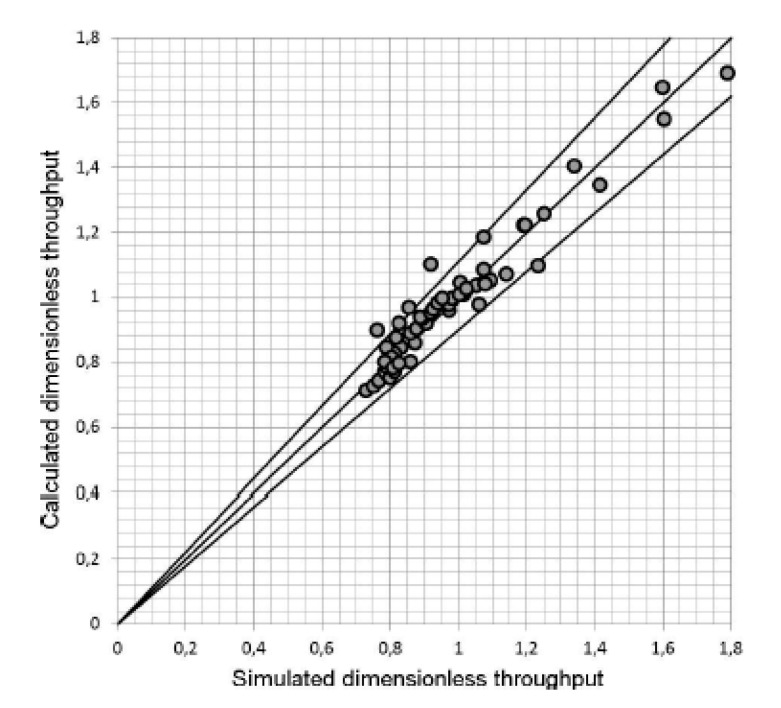
Polynomial regression of data—reprinted with permission from [23].

It is also possible to use DEM to show the dependence of flow rate on geometric and operating conditions in the solid-conveying zone [23]; authors address the problem of the influence of a series of characteristic parameters of the solid-conveying zone on the flow rate. The software used to perform DEM simulations is EDEM. At first, the excellent correspondence for LPDE between experimental and numerical flow rate values at different screw rotational speeds is observed. This is achieved through a proper testing of the material under extrusion, which allows to determine the exact value for both internal and external friction coefficients. Subsequently, authors perform a dimensional analysis to assess the influence of the main parameters, which are flow rate, screw diameter, screw speed, channel height, and the extension of the zone below the hopper. Finally, they determine the functional dependence of non-dimensional flow rate on the other non-dimensional parameters through a polynomial regression of numerical data (Figure 6).

The predictive capabilities of DEM are remarkable, and it is natural to think of an integration of this method with the numerical methods related to the compression and metering zones, usually studied through computational fluid dynamics (CFD) techniques.

Through this combined approach, a more accurate quantitative description of the extrusion process along the entire feed-zone length for PAM processes is expected.

### 2.2. Compression Zone

#### 2.2.1. Analytical Modeling

The first analytical work for the prediction of melting length in IM single-screw extruders was proposed by Tadmor in [28], developed according to the experimental data of Maddock and Street [29].

In [29], a series of studies on the dynamics of the melting process were carried out by means of the screw pulling-out technique. It consists of stopping the extrusion system, cooling it quickly, and, lastly, removing the external barrel. The state of the polymer in the screw vanes is thus clearly visible.

By cutting the polymer along the axial direction, it is possible to measure, vane by vane, both the solid and melt content, finding the local melting rate. Usually, in order to separate the solid zone from the molten one, the same material with a different pigment is added in the extrusion process [8,9,10].

On the base of their first pioneering studies, Maddock and Street found that the material melts only near the barrel surface because the polymer temperature is locally higher than its melting point. This thin molten layer shifts slowly towards the active flight, while the solid material is in contact with the passive flight. In this way, there is the formation of a melt pool where the molten polymer accumulates.

**Figure 7:**
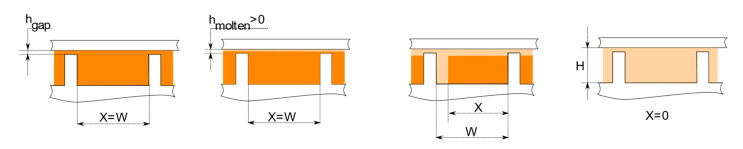
Maddock melting mechanism: W—channel width, H—channel height, h_gap_—radial clearance between screw flights and barrel, h_molten_—local molten layer thickness, X—local solid bed width.

This melting mechanism, namely the formation of a recirculating melt pool on the active flight while the solid bed is near the passive flight, is called the Maddock melting mechanism, and it has been noted for a large class of polymers used in the IM process. A schematic representation of this process is also given in Figure 7.

In the analytical study performed in [28], the channel is assumed to be unwound from the screw. Because of this assumption, the influence of screw curvature on flow and temperature fields is neglected. It must be pointed out that this assumption is justifiable only for extruders with a diameter sufficiently large, as in the case of BAAM and IM, but not in the case of MiE.

Tadmor assumes that the solid bed remains of rectangular cross-section while moving downstream is in a homogeneous state, that the local solid–melt interface is not varying in time, and that all thermodynamics properties are constants. Moreover, molten polymer is assumed as a Newtonian incompressible fluid.

The analytical solution proposed by Tadmor in [28] is based on some assumptions which are too strict for real applications in the field of IM and PAM processes. First, the constitutive relation chosen for polymer modeling is not representative of most real polymers’ rheological behavior; in fact, polymers generally exhibit a power-law and temperature-dependent behavior. For a given temperature, there is an exponential relationship between shear stress and shear rate. This dependence no longer exists in Newtonian fluids. In several further works [30,31], polymers have been modeled as a power-law fluid with an Arrhenius correction, so as to consider the relationship between rheological parameters and temperature.

It is important to underline that Tadmor in [32] also includes both non-Newtonian behavior and a molten layer thickness which is variable along screw axis, still under the common assumption of a constant downstream velocity for solid bed. Two additional problems of Tadmor’s theory are the assumptions of a linear temperature profile in the molten layer which forms between solid and barrel surfaces and that of zero pressure gradient.

In [30], the energy equation for a power-law fluid was solved analytically. Through a thermal balance on the interface between solid and melt polymer, the author then evaluates the melting rate. It is further demonstrated how this quantity decreases by increasing the gap between flights and screw, and how it is related to barrel temperature.

In general, the energy requested for polymer melting is given by two main contributions: the first is heat conducted from the heated barrel towards the polymer and the second is viscous heating. The latter consists of heat generated by viscous dissipation, and it is due to shear dependent behavior of the polymer in the molten layer. It is estimated that ca. 80% of the total heat required for polymer melting is given by viscous heating [7] for a standard IM single-screw extruder.

The relative importance of viscous heating and conduction heat transfer is defined by the Brinkman number (Br).

**Figure 8:**
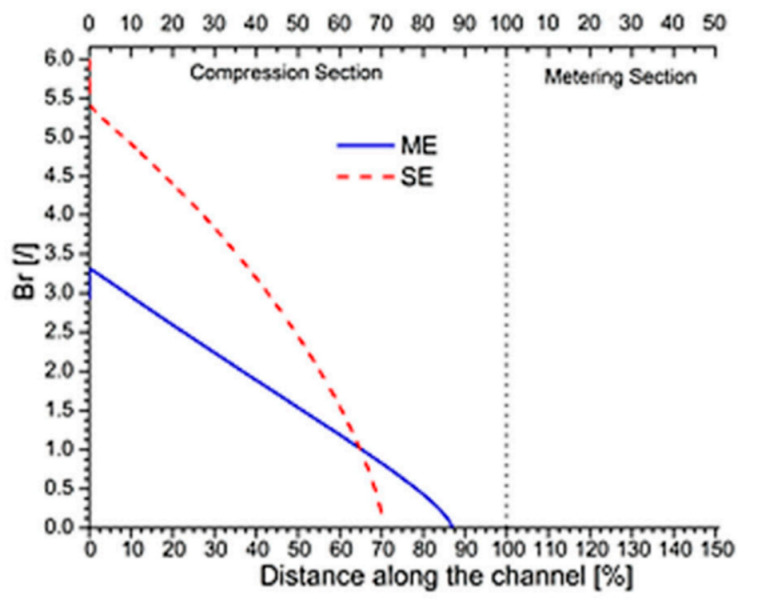
Brinkman number (Br) along screw channel direction for both the standard extruder used in the IM process (SE) and the mini-extruder (ME) used in the pellet extrusion-based process—reprinted from [7].

A very impressive study regarding the influence of both extruder dimensions and operating conditions on Br and melting rate was presented in [7]. In fact, the main purpose in [7] was to give a deep insight into pellet extrusion-based systems’ melting dynamics. It was demonstrated that in the case of MiE and applying the modified Tadmor model proposed in [32], lower values of Br characterize most of the screw length because the local value of shear rate is lower than in the case of an IM standard extruder. The opposite tendency was shown in the last few turns of compression zone. It is interesting that, for fixed operating conditions, rheological and thermo-fluid dynamics parameters for both solid and melt phases are the same as in [10], so the influence on Br is due only to screw dimensions. All previous conclusions are represented in Figure 8.

The main problem is that in [7], the modified Tadmor model [32] was implemented, which is valid for polymer flow in flat rectangular channels. Turning to PAM, this assumption is no longer valid for MiE because curvature cannot be neglected, but it can still be applied in BAAM process modeling because of the higher diameter/channel width ratio.

As mentioned earlier, the Maddock melting mechanism is the most observed, but it cannot be valid for all polymers and operating conditions.

In fact, melt pool can also form close to the passive flight; this is referred to as the Klenk melting mechanism [33] and it has been observed for PVC.

It is even possible that there is a melt pool both close to the active and passive flight, with thin melt layers near both the screw and barrel surfaces; in this case, the solid material is completely surrounded by molten polymer. In literature, this behavior is commonly known as the Lindt melting mechanism [31]. This melting mechanism was initially observed for PP.

As well as implementing this model for the first time, Lindt shows in [31] how the melting mechanism is strictly dependent on the transversal force acting on the solid polymer, where this force depends on the pressure gradient, already for the simple Newtonian case.

**Figure 9:**
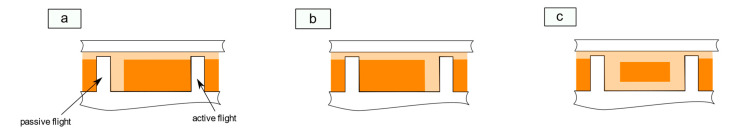
(**a**) Klenk; (**b**) Maddock; (**c**) Lindt melting mechanism.

Both melting mechanisms are represented in Figure 9.

Other works [34,35,36,37] determined the power consumption in the extrusion process in the compression zone. The power requested to melt a polymer can be calculated analytically if the wall shear stress is also known. Wall shear stress is derived by the knowledge of the flow field. Furthermore, wall shear stress is a function of sliding velocity and metal surface temperature [34].

Moreover, the gap between top of the flights and barrel is a region of flow field where there is significant viscous dissipation which has to be quantified in order to evaluate the total power needed for extrusion, i.e., as in the mathematical model proposed in [35].

#### 2.2.2. Computational Modeling

There are different limitations related to the analytical investigation of the compression zone; the most important is related to the fact that a specific melting mechanism must be assumed. In fact, it can vary with operating conditions, as shown in the previous section. Other problems are related to the highly non-linear nature of the coupled partial differential equations which must be solved.

Additionally, one of the most widespread assumptions in the analytical modeling of the compression zone is that of the no slip condition at the walls. As highlighted in [38,39], many materials of industrial interest exhibit wall slip. Some of these materials are PVC, HPDE, ceramic materials, foodstuffs such as meat and dough, filled polymers such as wood–plastic composites (WPC), and elastomers [38].

The problem of the wall slippage is handled by an analytical point of view in [37], where the equations for a one-directional Newtonian fluid in a straight channel were solved. Now, it is well known that flow behavior in all extrusion zones is at least two-directional and that fluid must be modeled properly as a non-Newtonian fluid whose rheological parameters are a function of temperature. In [38,39,40], the problem of modeling wall slipping materials during extrusion process in IM single-screw extruders was addressed through CFD.

We can use CFD simulations to overcome the analytical difficulties related to the highly non-linear and coupled nature of the equations; by solving conservation principles, namely mass, momentum, and energy equations together with an appropriate constitutive relation for the material being extruded, there is no longer the necessity to know a priori the melting mechanism. Moreover, the influence of the slippage parameters can be investigated for the materials listed above.

In addition, CFD simulations allow to study control volumes of complex shapes, such as the curved geometries involved in the modeling of real extruding vanes of MiE; this can be used to relax the widely adopted assumption of a straight rectangular channel and to develop predictive computational models.

Authors in [8] lead both numerical analysis with PELDOM software and experimental investigations through the screw pulling-out technique for an IM single-screw extruder. Their purpose was to analyze the melting mechanism and pressure development during the melting of ABS in a straight channel.

In performing the experiments, black pigmented ABS was added with the purpose of identifying where the material was in the molten state. It was found that the melting process follows, both analytically and numerically, the Maddock melting mechanism, but the numerical results show a larger melting rate.

The same authors in [10] used PELDOM software to analyze the melting process dynamics and evolution of the pressure profile along the screw axis for ABS extrusion. It was demonstrated through a parametric study that increasing screw temperature leads, for a given axial position, to a decrease in the content of solid material and to an increase of predicted pressure.

**Figure 10:**
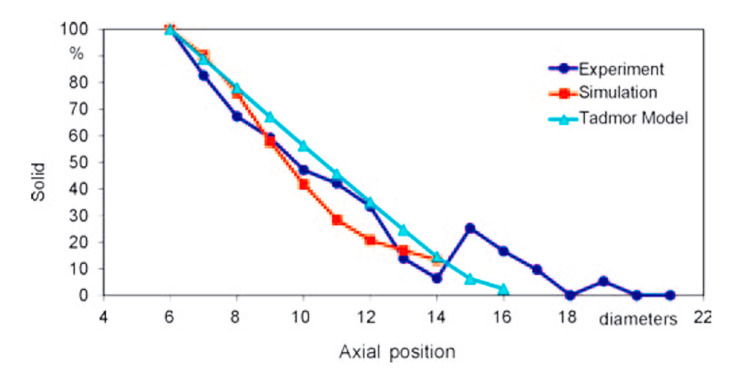
Experimental, CFD, and analytical predictions of the solid content in screw vanes of an IM extruder—reprinted with permission from [10].

These profiles are also largely dependent on the viscosity of the polymer. Increasing viscosity leads to a net increase of the axial pressure and to a decrease in the solid content. A similar behavior was found decreasing the flow rate. It is remarkable that in their last work [10], the authors implemented a direct comparison of experimental, numerical, and analytical results (Figure 10); the latter are extracted through Tadmor’s model.

Figure 10 shows how the modified Tadmor model, which also accounts for the non-Newtonian behavior of molten polymer, over-predicts the melting rate. The same conclusion can be stated for simulations, in the axial positions from 9 to 13 diameters from the hopper.

In [20], the IM process in a straight channel made of both compression and metering zones is simulated with CFD software Fluent by comparing numerical results with experimental and numerical results given in [10], finding a good agreement in terms of melting rate. However, there is still a different behavior of pressure in the metering zone. The numerically predicted pressure gradient is positive along the entire metering zone, while in experiments (and in other studies performed solely for the metering zone, i.e., the analytical results proposed in [36]), it is negative. The reasons for this different behavior must be better understood.

### 2.3. Metering Zone

#### 2.3.1. Analytical Modeling

Once the polymer is completely molten, it must be transferred towards the die in the case of an IM extrusion system, or towards the extruding nozzle in the case of PAM extrusion processes.

In the framework of IM, the first analytical work modeling molten polymer transport is [41]; the isothermal flow of a Newtonian fluid in screw vanes was studied, finding a solution for the two-dimensional pure drag flow.

In [42], the problem was solved for a Newtonian fluid, by determining both the flow rate and pressure distribution under the assumption of a channel of infinite width. The ratio between width and height of the channel is usually referred to as the channel aspect ratio.

These two assumptions, namely Newtonian fluid and an infinite aspect ratio of an unwound screw extruder channel, ensure that, in the case of a one-dimensional flow, the partial differential equation reduces to an ordinary one [42]. This equation, together with the boundary condition of a moving top plate (namely the barrel in IM and PAM processes) with a stationary bottom surface, gives the well-known Couette flow equations. By further assuming that the flow rate in the die is proportional to the associated pressure drop, it has been found that the pumping characteristics in IM are linear both for screw and die. The operating point is given by the intersection of these two characteristics. In [43], these findings are integrated by considering the solution for the two-dimensional problem, always in the case of Newtonian fluid. The author gives some correction coefficients for both pressure and drag flow, so as to find the right flow rate for the extrusion through channels of semi-elliptical or rectangular cross-section.

Now, let us deal with the power demand in the metering zone. This is made up of both the power requested for extrusion process and power dissipated in the radial gap between flights and barrel. In [44], an analytical solution for the isothermal flow of Newtonian fluids in unwound screw channels which also considers the power consumption related to leakage flow in the gap between flight tips and barrel is proposed.

In [45], the functional dependence of power consumption in the radial clearance between flight and barrel on both flight width and screw length is widely described, showing that in the case of Newtonian fluids, there is an optimal value for radial clearance which allows the designer to minimize this contribution to the total power consumption requested for extrusion.

The problem of the previous studies lies naturally in the Newtonian fluid assumption.

To solve this problem, a first way is to consider a mean value for viscosity when this parameter cannot be considered as a constant along the entire screw length [44], but this approach cannot be exhaustive of the real behavior of the polymer, which is widely non-Newtonian with rheological parameters which are also temperature dependent. In fact, in polymer extrusion, it is mandatory to consider the constitutive relation between shear stress, shear rate, and temperature. One study attempted to also estimate leakage flow in the case of an isothermal flow of a power-law fluid [46].

On the other hand, the power requested for the extrusion process is strictly related to an accurate calculation of flow field, upon which relies the calculus of wall shear stress and pressure development.

In [47,48], the equations describing the behavior of an isotropic, incompressible, and inelastic fluid are derived through a phenomenological theory for macro-rheology. These works assume that stress tensor is a function of kinematic matrix and some material constants.

In [49], the stationary, laminar, isothermal Couette flow of a non-Newtonian fluid was solved analytically. The relationship between shear stress and shear rate was expanded through a polynomial series and the solution was found considering a shear rate contributing up to third order. The solution can be obtained for an arbitrary number of terms, but the coefficients which appears in the polynomial expansion must be determined experimentally. Similarly, a two-coefficient-based problem for the pseudoplastic material model can be solved. Solutions are proposed both for Rabinowitsch and the general pseudoplastic constitutive relations.

A similar model for the study of a fluid modeled through a Rabinowitsch constitutive relation, but this time in non-isothermal flow conditions, was proposed in [50].

The issue of the evaluation of flow field for non-Newtonian fluids has been also addressed analytically in [51] with the possibility of employing non-Newtonian fluids in thrust bearings, and in [52] for generalized Couette flows. An analytical solution for the isothermal and one-directional flow of a power-law fluid in a channel with varying height in downstream direction was derived in [51].

The assumption of an isothermal flow was removed in [53], where the analytical solution to the thermo-fluid dynamics problem of the transport of molten material was given for a power-law fluid in presence of viscous dissipation.

In [49,51,53], it is also assumed that the channel is flat and characterized by an infinite aspect ratio.

Moving on to PAM processes, a single-screw extruder for MiE is generally smaller than that used for IM and BAAM; for this reason, both curvature effect and real geometry of the channel must be considered, as seen in the previous section.

In contrast to [43], in [54], the problem for non-Newtonian fluids in finite aspect ratio channels was solved and the effect of lateral screw flights on flow field was addressed. There it was underlined as already in the simplified case of an incompressible, isothermal flow of a non-Newtonian fluid in a finite aspect-ratio rectangular channel and with motion imposed to the barrel, that the problem is modeled by highly non-linear partial differential equations for which an analytical solution no longer exists.

Similarly, in [55], an analytical model for the flow of Newtonian fluids in finite aspect ratio channels was proposed, and both the pressure-flow rate relation and flow field along the channel height for corn syrup, which can be assumed as a Newtonian fluid, were well predicted. In [36,56,57,58,59], the authors refer to correction factors for pressure and drag flow, as suggested in the case of Newtonian fluids in [43]; this is a really simple solution for taking into account the limited aspect ratio, and so the influence of both trailing and pushing flights on the flow field. Moreover, it has been shown that at least for the drag flow correction factor, there are no significant differences between the values measured for various polymers and the theoretical values calculated for Newtonian fluids [60,61]. However, there is a lack of information on helix angle influence on the flow field. This effect was addressed in [62], where a complex theory involving channel curvature was developed. We will see later other works where the problem of defining governing equations in frame references different from the Cartesian one were addressed.

There are a few studies which face the solution of the problem of modeling non-Newtonian flow behavior by an analytical point of view, and it has been widely shown that these studies are based on restrictive assumptions such as isothermal flow through flat rectangular ducts of infinite aspect ratio.

In [63], the analytical solution for the isothermal, incompressible flow of power-law fluid is proposed, where the main task is to optimize geometrical features of the extrusion screw, by trying to link optimal values to rheological parameters, under the assumption of considering a flat channel with infinite aspect ratio. One of the most distinctive results lies in the fact that the optimum helix angle is only a function of power-law index. Validation has been done with the experimental results proposed in [64], finding good agreement only if the power-law index is greater than 1/√2. Moreover, it is assumed that both channel curvature and flights have no effect on the results and that rheological parameters are independent of temperature. Despite this work being of great importance for giving design optimum indications for a limited range of pseudoplastic fluids, these two latter assumptions are too restrictive for the description of the transport of molten polymer in the metering zone of a MiE, where curvature cannot be neglected.

**Figure 11:**
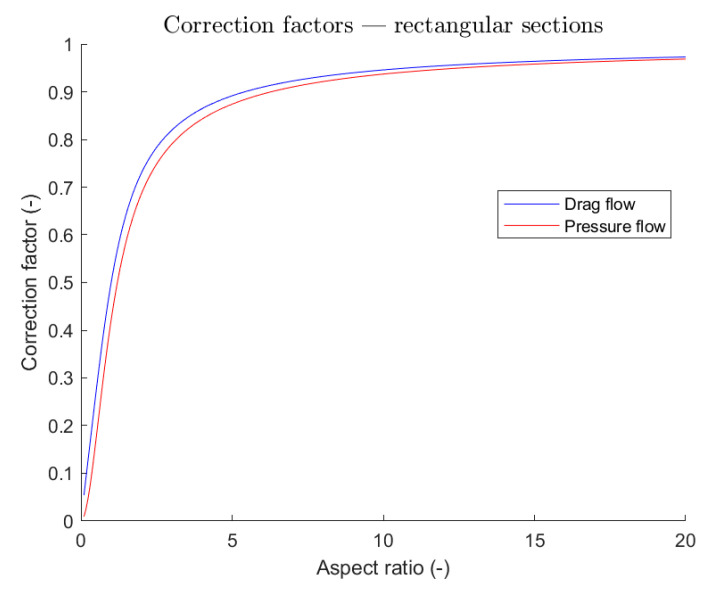
Correction factors for rectangular channels for a given aspect ratio (width to height ratio).

There are a few studies in literature which try to take into account channel curvature in metering section through correction factors [62] (Figure 11) or achieving the solution in a coordinate system different from the Cartesian one [57,65].

The first work which analyses the influence of helix angle and curvature on both flow and pressure fields is [62]. In fact, helix angle changes with radial distance and this variation has been neglected in previous works. A limit of this study is that only Newtonian fluids was discussed.

Various works, such as [32,35,65], look for analytical and numerical solutions in order to consider channel curvature, in the general case of non-Newtonian fluids. In [57], an analytical solution is proposed in the case of an Ellis fluid, which is a generalization of the power-law constitutive relation. Another way to consider effects related to channel curvature on flow, pressure, and temperature fields is to study the problem in helical coordinates, as in [66,67,68,69,70]. This approach would allow to consider both curvature and helicity of the channel for a single-screw extruder, despite their application being in the field of IM or PAM.

There are different helical coordinate systems which have been suggested in literature: a physical basis of unit vectors in radial, helical, and axial direction could be used, starting from a cylindrical frame of reference [68], or the coordinate system can be set up starting from a Cartesian one [71].

Finally, as in the case of compression zone, the widely used assumption of no-slip condition at boundary/polymer interfaces must be discussed.

Wall slippage depends on the single-screw extruder geometry, polymer considered, surface properties, both intensity and direction along which pressure gradient acts, shear stress, and shear rate [72].

Physically, by gradually increasing shear stress, there is a transition from no-slip condition to an intermediate situation between no-slip and slippage. By increasing the shear stress above a threshold, there is wall slip condition.

Both [72,73] show analytical solutions for Newtonian fluids, by distinguishing that what happens in the case of non-dimensional shear stress where slippage begins is more or less than unity. This analysis leads to the conclusion that slippage can be unilateral, which means that slippage can occur only on a single surface, namely barrel or screw surface, or bilateral. The analytical expressions given in this work link non-dimensional flow rate to non-dimensional pressure gradient in all the possible operating conditions of a single-screw extruder in the metering zone. Results are validated in [72] by means of FEM simulations, with a good overall agreement.

The non-Newtonian behavior under wall slippage is analyzed by the same authors in [74] under the assumption of one-dimensional isothermal flow. It is shown as pumping characteristics of IM extrusion process deviate from linearity because of both pseudoplastic nature of processed material and wall slip; in fact, linear behavior was predicted for Newtonian fluids under no-slip condition [42].

We can conclude that analytical modeling of melt flow in screw vanes is a complex task, for which a variety of assumptions have been used in the past decades.

All the assumptions related to the flat plate theory, where curvature is neglected, cannot be used in modeling the MiE, but, if channel height to diameter ratio is low enough, it can be still used to estimate BAAM process performances.

Wall slipping, non-Newtonian fluid behavior, and non-isothermal conditions lead to a system of non-linear partial differential equations, for which it is more appropriate in general to use computational methods.

#### 2.3.2. Computational Modeling

Several numerical schemes have been proposed to solve the fluid dynamics problem of molten polymer transport toward the die in IM literature.

Among the main advantages related to numerical modeling of the extrusion process, there is the possibility to consider geometrical features which are too complex to model by an analytical point of view, especially curvature and real cross section shape (with finite aspect ratios).

As an example, a finite difference scheme for the solution of the flow in a channel with finite aspect ratio was proposed in [75]. Results are in good agreement with the analytical solution proposed in [55], already for a coarse mesh. The importance of the study presented in [75] is related to the possibility to perform analysis for non-rectangular cross-sections, such as that normally found in a single-screw extruder. This deviation from the ideal rectangular section is due to the fillets at the bottom of screw flights. By performing numerical simulations, there is no longer the need to consider the correction factors for pressure and drag flows represented in Figure 11 or the shape factors introduced in [42]. The main drawback of [75] is to consider the isothermal flow of a Newtonian fluid.

There is a wide range of numeric schemes used in literature for melt flow modeling. In [76], the Lattice gas automata method was used, while in [77], flow field in the metering zone under isothermal conditions was investigated via the Lattice–Boltzmann method, finding in the latter a good agreement with the analytical solution proposed in [61].

The main difference between numerical results of simulations conducted in [76,77] lies in the fact that while in the former some unrealistic eddies near the corners of the flight are predicted, in the latter these eddies are absent. These eddies may occur in the metering zone and both their dimensions and their influence on the flow field have been widely studied in [78] by means of OpenFOAM; particles which are involved in these eddies, also referred to as Moffatt eddies, are characterized by a residence time two times larger than that of the mean flow in screw vanes, already in the Newtonian case. We must consider that polymers in metering zones are subject to a temperature well above melting point and they may run in local thermal degradation, from which defects in the final product can arise. Polymer degradation is enhanced if residence time is too high.

The residence time of the particles involved in Moffatt eddies grows up to three times higher than mean polymer residence time if non-Newtonian fluid behavior in the duct is considered. In any case, Moffatt eddies can be totally avoided in screws where fillets near flights’ roots are large enough; in general, the SPI recommendation is to manufacture screws which have fillets’ radii greater than half of channel height [79]. This empirical rule is reasonable because it allows to completely avoid Moffatt eddies in the flow field in the metering zone also in non-Newtonian cases [78].

We also saw in the previous section that the power requirement in metering zone has been studied analytically under the assumption of Newtonian fluid, which is not the case in polymer extrusion regardless of the specific manufacturing process.

In [80], experimental results are given for corn syrup and the analytical model proposed in [55] gives good correspondences, but it is still limited to considering Newtonian fluids.

Griffith, in [64], also elaborates a numerical method for the solution of the flow problem in non-Newtonian cases; screw is assumed in motion, while barrel is stationary, that is the real kinematic condition.

Even in the assumption of an infinite aspect ratio, a numerical model where Arrhenius correction is used, so as to consider the dependence of both consistency index and power-law index on temperature, is developed in [64]. The conservation equations of mass, momentum, and energy are then solved numerically. Results in terms of non-dimensional pumping characteristics for the non-Newtonian case show a clear deviation from the linear behavior predicted in [42], for Newtonian fluids, already discussed in the previous section.

In [65], the effect of curvature of the channel on the flow rate and on the viscous dissipation was shown by considering a two-dimensional, isothermal flow of a power-law fluid. Despite the effect of curvature having been considered, the effects related to the screw flights were neglected. Radial pressure gradient was neglected because of the very low Reynolds number. There was an overall agreement between the non-dimensional flow rate-pressure relationship found numerically in [64] with the results suggested in [79], where channel was considered flat both for Newtonian and non-Newtonian cases. In other words, results given for a theoretically infinite screw radius match that of flat plate theory. A remarkable influence of aspect ratio on viscous dissipation was also observed.

We have seen how the effect of both pushing and trailing flights can also be modeled using a different frame of reference. The same approach is possible if the same equations are formulated in the new system.

In [66], a FEM solution was given with the purpose of analyzing a fully developed, isothermal flow of a Newtonian fluid, using a helical frame of reference. This model was then validated with particle tracking technique in [80].

A similar formulation in helical coordinates, but with a functional which takes in account both velocity and temperature was proposed in [68].

An assumption which is very common to most of analytical and numerical models is that of a motion given to the barrel. These are also referred to as reverse kinematics conditions.

Investigation of the accuracy given by this kinematics assumption is addressed in different works [81,82,83,84].

It has been shown both analytically and experimentally that the assumption of giving the motion to the barrel is justifiable [82]; in particular, the model for the unwound channel with motion given to the barrel and with stationary screw is reasonable if the screw diameter is larger than 10× channel height. In [83], an analytical model, validated through FEM simulations, was given taking into account the general case of a non-isothermal flow. Results are identical for the two cases.

Temperature field is also the same in the two kinematic conditions, and this has been demonstrated by means of the FVM method [84].

In all studies discussed up to now, the no-slip condition was postulated for the modeling of the metering zone. As in the compression zone, the effect of wall slippage must be quantified to deal with different classes of materials.

The assumption of isothermal flow was neglected in [85], where a FEM 3D model was developed for the prediction of the temperature field development in screw channels under wall slippage.

In [40], CFD analysis was carried out with the software Polyflow for a non-Newtonian fluid. Authors determined flow and pressure fields, and, consequently, pumping characteristics and their dependence on the parameters which rule wall slippage. Finally, they led a similar analysis for the die of an IM single-screw extruder, determining the operating point. Thereby, it was shown how the operating point is influenced by the parameters involved in wall-slip phenomena.

## 3. Conclusions

The most important works related to the IM process modeling have been reviewed with the purpose of verifying what assumptions still hold in the study of PAM processes, namely BAAM and MiE.

It has been shown that some of them must be removed if one wants to extend these models to single-screw extrusion, especially in the case of MiE because of the different scale.

By studying the thermo-fluid dynamic problem of the flow through a rectangular duct, errors are expected to be greater than for IM, when it comes to MiE; it has been shown in literature that this error increases as long as the ratio between channel width and screw diameter increases.

Dimensions can also affect melting rate; melting length is higher than in a standard IM extruder because of the lower Br number, thus operating conditions must be chosen carefully if one desires to avoid defects related to incomplete and/or not homogenous melting in the final product. These conclusions must be proven by means of a model for the compression zone capable of performing analyses which consider the influence of curvature on melting profile.

In addition, the assumption of a stationary screw with moving barrel becomes questionable if the diameter is not larger than 10× channel height, so this aspect must be considered in performing CFD simulations and elaborating analytical models.

Moreover, the no slip condition at both barrel and screw walls has to be relaxed for a large range of materials of industrial interest. Because it is analytically difficult to take into account the complex polymer rheology, CFD is mandatory. An accurate characterization of the polymers involved in extrusion process is requested, especially when momentum exchange between pellets through DEM must be modeled.

From this perspective, the numerical solution which has the major potential to give the best results in PAM modeling involves a mixed DEM–CFD approach.

**Author contributions:** Conceptualization, A.P., G.P. and M.D.d.T.; writing—original draft preparation, A.P.; writing—review and editing, A.P., G.P. and M.D.d.T.; visualization, A.P.; supervision, G.P. All authors have read and agreed to the published version of the manuscript.
